# Tibial tubercle involvement in tibial plateau fractures is associated with nonunion, reoperation, and flexion deficits

**DOI:** 10.1007/s00590-026-04808-9

**Published:** 2026-07-29

**Authors:** Robert G. Hernandez, David Dallas-Orr, Noah L. Lyndall, Tyler E. Lockwood, Shannon Tse, Gillian L. S. Soles, Ellen P. Fitzpatrick, Mark A. Lee, Sean T. Campbell, Augustine M. Saiz

**Affiliations:** 1 School of Medicine, University of California, Davis, Sacramento, USA; 2 Department of Orthopaedic Surgery, University of California Davis Medical Center, Sacramento, USA

**Keywords:** Tibial plateau fractures, Tibial tubercle, Range of motion, Orthopedic surgery, Rehabilitation

## Abstract

**Purpose:**

Tibial tubercle involvement (TTI) in tibial plateau fractures (TPF) is understudied. We evaluated whether TTI is associated with major postoperative complications and range-of-motion (ROM) recovery after operative TPF compared with isolated TPF (iTPF).

**Methods:**

We performed a retrospective cohort study of operatively treated TPFs from 2014 to 2025. TTI was identified using radiographs, CT scans, and operative reports. Primary outcomes were knee ROM (flexion and extension) at 6 weeks, 12 weeks, and 1 year, as well as postoperative complications including reoperation and nonunion. Covariates included demographics, injury and operative characteristics, complications, and postoperative weight-bearing/immobilization status.

**Results:**

Among 132 TPFs, 38 (28.6%) had TTI and 94 (71.2%) were iTPF, with similar baseline age, BMI, sex, and ISS. TTI injuries were more frequently Schatzker VI and open, whereas iTPF were more commonly Schatzker II. Weight-bearing prescriptions were similar, but immobilization was more frequent with TTI. Compared with iTPF, TTI was not associated with significantly different flexion at 6 weeks, but was associated with lower flexion at 12 weeks (adjusted difference − 27.31°, 95% CI [− 37.73, − 16.88]; *p* < 0.001) and at 1 year (adjusted difference − 19.94°, 95% CI [− 35.41, − 4.48]; *p* = 0.011). Extension recovery did not differ over time. In the cohort with ≥ 3-month follow-up, TTI was associated with higher reoperation (39.4% vs. 11.2%, *p* = 0.001), nonunion (15.2% vs. 2.5%, *p* = 0.022), and culture-positive infection requiring reoperation (30.3% vs. 10.0%, *p* = 0.011). In adjusted analysis, TTI remained associated with any reoperation (OR 3.95, 95% CI [1.36, 11.48]; *p* = 0.012).

**Conclusion:**

TTI was associated with higher rates of nonunion and reoperation after operative treatment of TPF, and with reduced flexion ROM at both 12 weeks and 1 year. However, these findings should be interpreted in the context of greater injury severity and more frequent postoperative immobilization in the TTI cohort.

## Introduction

Tibial plateau fractures (TPF) represent about 1% of all fractures and 8% among older populations [1]. The articular anatomy of the proximal tibia is complex in nature; therefore, restoring knee joint congruity after TPF is an important modifiable risk factor for reestablishing knee function. This is because residual articular step-off or gap and malalignment significantly increase the risk of posttraumatic osteoarthritis and conversion to total knee arthroplasty (TKA) [2–4]. Depending on the fracture pattern, various surgical methods can be tailored to achieve postoperative goals such as restoring articular congruity, anatomical alignment, and stable fixation that allows for an early range of motion. TPFs may also involve the tibial tubercle, which is the bony prominence located on the anterior aspect of the proximal tibia where the patellar ligament inserts onto and must be stabilized due to its impact on the extensor mechanism of the knee [5, 6]. Therefore, improving stability and functionality of the knee requires stabilizing the tibial tubercle in managing tibial plateau fractures [6]. This stabilization may be achieved through various surgical techniques modified to the fracture pattern with the postoperative goals of restoring articular congruity, anatomical alignment, and stable fixation that facilitates early range of motion [6–8].

Although tibial tubercle involvement may significantly impact outcomes after tibial plateau fractures, there’s still limited research examining how it affects postoperative recovery. Prior studies on tibial plateau fracture management have primarily concentrated on surgical and rehabilitative methods, with less attention to the difficulties and potential clinical implications of tibial tubercle involvement [7, 8]. Therefore, the purpose of this study was to investigate the impact of tibial tubercle involvement (TTI) on postoperative complications and quantify the potential effects on range of motion (ROM) compared to cases with isolated tibial plateau fractures (iTPF). We hypothesized that tibial tubercle involvement (TTI) would be associated with increased postoperative morbidity, specifically with higher rates of reoperation and nonunion, as well as worse postoperative knee motion compared to isolated tibial plateau fractures (iTPF).

## Patients and methods

**Fig. 1:**
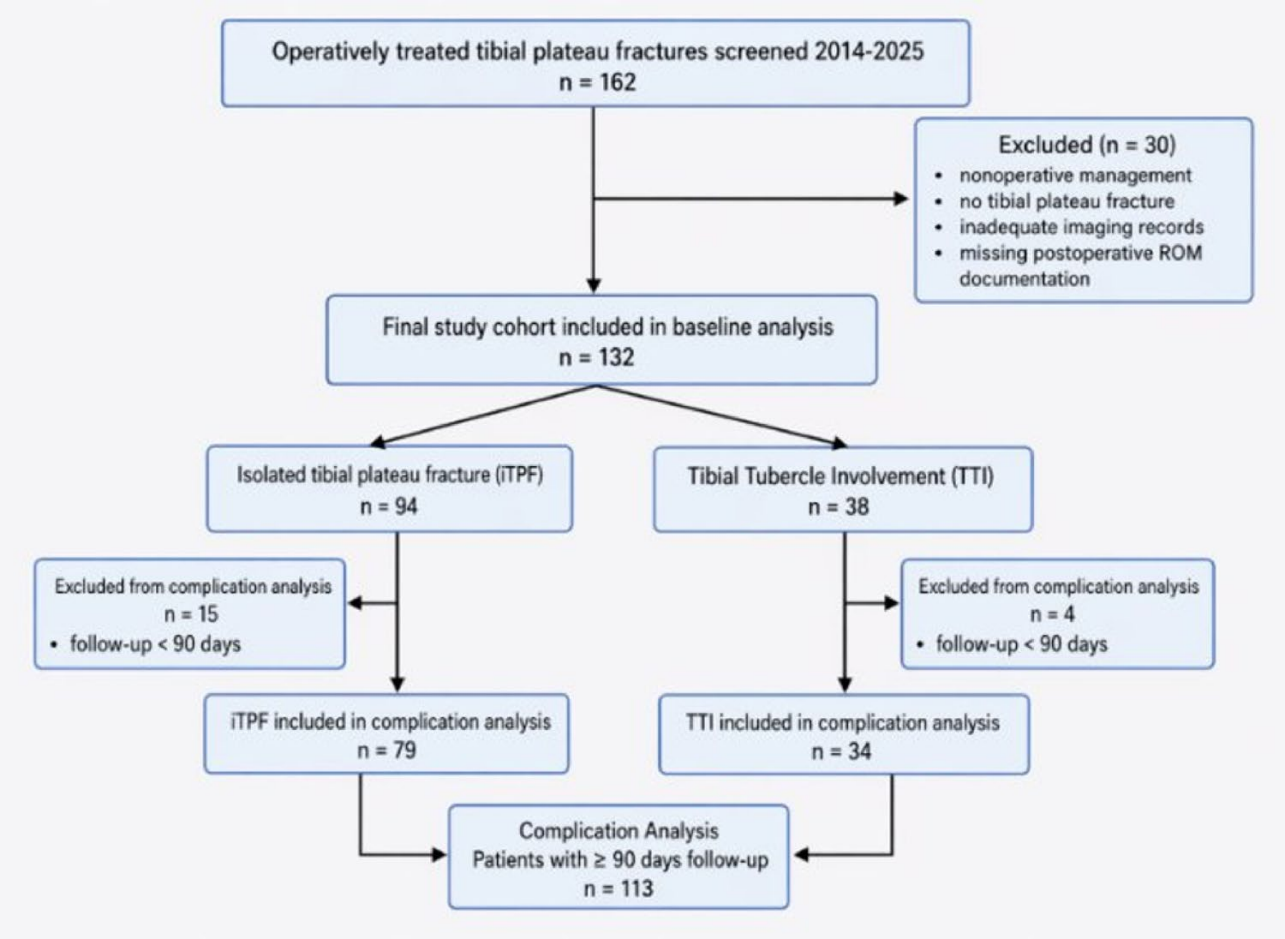
Patient flow diagram demonstrating cohort selection, exclusions, and derivation of the complication analysis cohort

Following institutional board review approval, a retrospective study was conducted on patients who sustained a TPF between 2014 and 2025. Patients were screened for concomitant tibial tubercle involvement (TTI), which was determined through review of radiographic images, CT scans, and operative notes (Fig. 1). TTI classification was performed by two independent reviewers with disagreements evaluated by a third reviewer. A separate tibial tubercle fragment was defined as a distinct fracture line through the anterior tibial tuberosity, resulting in a cortically isolated fragment visible on two-dimensional CT axial imaging or lateral plain films. Postoperative knee ROM was extracted from clinical notes at 6-week, 12-week, and 1-year follow-up visits. Following surgery, immobilization using either a straight leg brace or a hinged knee brace locked in extension was performed at the ultimate discretion of the treating surgeon. Weight-bearing progression and ROM advancement were likewise determined by the treating surgeon and were not standardized across the study period. Patients without active records of ROM in clinic notes were excluded from the analysis.

Patient demographics, including age, sex, body mass index, smoking status, and history of diabetes, tobacco use, and autoimmune disease, were collected through review of medical charts. Injury characteristics included Schatzker classification, open fracture status, and polytrauma status, which was defined as an Injury Severity Score greater than 15. Surgical details, including the specific approach (anterolateral, medial), method of fixation (plating, screw fixation), operative duration, estimated blood loss, and intraoperative complications, were extracted from operation notes and supplemented by review of postoperative radiographs. Postoperative complications, including need for revision surgery, nonunion, deep infection, and wound dehiscence, were recorded. Nonunion was characterized by the radiographic presence of a fracture line that persists for 9 months without signs of healing for 3 months or the necessity for any secondary surgical intervention to achieve union of healing. Reoperation was defined as any unplanned return to the operating room for complications directly related to the index fracture or its internal fixation, including but not limited to: surgical debridement for infection, revision for fixation failure or nonunion, and removal of symptomatic hardware.

Linear mixed-effects models with random intercepts for patient were used to evaluate flexion and extension ROM across the 6-week, 12-week, and 1-year follow-up visits. Fixed effects included tibial tubercle involvement, time, the tibial tubercle involvement × time interaction, immobilization status, open fracture status, and Schatzker VI fracture pattern. Chi-square and Fisher exact tests were used to compare postoperative weight-bearing and immobilization status between cohorts. Postoperative complications were compared in the subset of patients with at least 3 months of follow-up using Fisher exact tests. Adjusted logistic regression was then used to evaluate the association between tibial tubercle involvement and any reoperation in that complication cohort. To reduce overfitting, the adjusted model was limited to tibial tubercle involvement, open fracture status, and Schatzker VI fracture pattern. All statistical analyses were performed in SPSS (IBM, Chicago, IL) and a *p* value of < 0.05 was considered statistically significant.

## Results

A total of 132 patients were included, of whom 94 (71.2%) sustained isolated tibial plateau fractures (iTPF) and 38 (28.6%) sustained fractures with tibial tubercle involvement (TTI). Patients in the tibial tubercle cohort were significantly more likely to present with Schatzker VI fractures compared to the isolated tibial plateau group (65.8% vs. 24.5%, *p* < 0.01). Conversely, isolated tibial plateau fractures were more frequently classified as Schatzker II (27.7% vs. 13.2%, *p* < 0.01) (Table 1). Additionally, the rate of open fracture was significantly higher among patients with tibial tubercle involvement compared to isolated tibial plateau fractures (13.2% vs. 2.1%, *p* = 0.02). No significant differences were observed between groups with respect to age, sex, BMI, or the other comorbidities of diabetes, autoimmune disease, substance abuse, or ASA classification. The cohort had an average age of 47.4 years and consisted of 67 (50.3%) males. The mean Injury Severity Scores of the TTI and iTPF groups did not show significant differences (TTI 10.7 ± 9.7; iTPF 8.7 ± 7.8; *p* = 0.22).

**Table 1:** Baseline demographics and clinical characteristics of patients with isolated tibial plateau fractures versus tibial plateau fractures with tibial tubercle involvement

	Overall	iTPF	TTI	*p* value
Patients	132	94 (71.2)	38 (28.6)	
Age (mean ± SD)	47.4 ± 14.9	48.3 ± 14.4	45.1 ± 15.9	0.26
BMI (kg/m^2^) (mean ± SD)	29.7 ± 7.0	29.9 ± 7.5	29.4 ± 5.5	0.74
*Sex*				
Male	67 (50.5)	47 (50.0)	20 (52.6)	0.78
Female	65 (49.2)	47 (50.0)	18 (47.4)	
*History of tobacco use*				
Yes	37 (15.9)	21 (22.3)	16 (42.1)	0.02*
No	96 (55.3)	73 (77.7)	22 (57.9)	
*Substance abuse*				
Yes	24 (11.4)	15 (16)	9 (23.7)	0.30
No	109 (59.8)	79 (84)	29 (76.3)	
*Diabetes*				
Yes	9 (4.5)	6 (6.4)	3 (7.9)	0.72
No	124 (66.7)	88 (93.6)	35 (92.1)	
*Autoimmune disease*				
Yes	10 (6.1)	8 (8.5)	2 (5.3)	0.72
No	123 (65.2)	86 (91.5)	36 (94.7)	
*ASA*				
1	17 (9.1)	12 (12.8)	4 (10.5)	0.07
2	71 (42.4)	56 (59.6)	15 (39.5)	
3	41 (17.4)	23 (24.5)	18 (47.4)	
4	3 (2.3)	3 (3.2)	1 (2.6)	
*Polytrauma*				
Yes (ISS > 15)	7 (3.8)	5 (5.3)	2 (5.3)	1
No	126 (67.4)	89 (94.7)	36 (94.7)	
*Open fractures*				
Yes	7 (1.5)	2 (2.1)	5 (13.2)	0.02*
No	126 (69.7)	92 (97.9)	33 (86.8)	
*Schatzker*				
1	6 (4.5)	6 (6.4)	0 (0)	< 0.01*
2	31 (19.7)	26 (27.7)	5 (13.2)	
3	9 (6.8)	9 (9.6)	0 (0)	
4	9 (6.1)	8 (8.5)	0 (0)	
5	29 (15.9)	21 (22.3)	8 (21.1)	
6	48 (17.4)	23 (24.5)	25 (65.8)	
Follow-up time (years)	1.25 ± 1.52	1.32 ± 1.68	1.10 ± 1.06	0.23

ASA = American society of anesthesiologists score; BMI = body mass index; ISS = injury severity score, iTPF = isolated tibial plateau fracture, TTI = tibial tubercle involvement

* indicates statistical significance at *p* ≤ 0.05

The postoperative weight-bearing instructions were not significantly different between the 2 cohorts (*p* = 0.154). However, immobilization was significantly more common in the TTI group than in the iTPF group (29% vs. 5%, *p* = 0.001) (Table 2). In longitudinal analysis utilizing a mixed-effects model, flexion recovery differed by fracture group over time (overall TTI × time interaction, *p* < 0.001) (Table 3). Compared with iTPF, TTI was not associated with significantly different flexion at 6 weeks (adjusted difference − 1.26°, 95% CI [− 11.62, 9.11]; *p* = 0.812), but was associated with significantly lower flexion at 12 weeks (adjusted difference − 27.31°, 95% CI [− 37.73, − 16.88]; *p* < 0.001) and at 1 year (adjusted difference − 19.94°, 95% CI [− 35.41, − 4.48]; *p* = 0.011). Extension recovery did not differ significantly over time between groups (overall TTI × time interaction, *p* = 0.514).

**Table 2:** Comparison of postoperative weight-bearing and immobilization status between isolated tibial plateau fractures and tibial tubercle involvement

	iTPF (%)	TTI (%)	*p* value
*Weight-bearing status*			
NWB	83 (84)	35 (95)	0.154
PWB/TDW/TTW	16 (16)	2 (5)	
*Immobilization status*			
Not immobilized	94 (95)	27 (71)	< 0.01*
Immobilized	5 (5)	11 (29)	

* indicates statistical significance at *p* ≤ 0.05

iTPF = isolated tibial plateau fracture, NWB = non-weight bearing, TDW = touch down weight bearing, TTI = tibial tubercle involvement, TTW = toe touch weightbearing

**Table 3:** Mixed-effects model comparison of postoperative range of motion between isolated tibial plateau fractures and tibial tubercle involvement

Model result	ROM estimate	95% CI	*p* value
*Flexion mixed-effects model*			
Overall TTI × time interaction			< 0.001*
Adjusted between-group difference at 6 weeks (TTI - iTPF), degrees	− 1.26	[− 11.62, 9.11]	0.812
Adjusted between-group difference at 12 weeks (TTI - iTPF), degrees	− 27.31	[− 37.73, − 16.88]	< 0.001*
Adjusted between-group difference at 1 year (TTI - iTPF), degrees	− 19.94	[− 35.41, − 4.48]	0.011*
*Extension mixed-effects model*			
Overall TTI × time interaction			0.514
Adjusted between-group difference at 6 weeks (TTI - iTPF), degrees	0.34	[− 2.11, 2.79]	0.787
Adjusted between-group difference at 12 weeks (TTI - iTPF), degrees	− 0.89	[− 3.38, 1.59]	0.481
Adjusted between-group difference at 1 year (TTI - iTPF), degrees	0.38	[− 3.37, 4.12]	0.844

* indicates statistical significance at *p* ≤ 0.05

iTPF = Isolated Tibial Plateau Fracture, ROM = Range of Motion, TTI = Tibial Tubercle Involvement. Linear mixed-effects models were used to assess longitudinal ROM recovery while accounting for repeated measurements within patients. Fixed-effects models included TTI, follow-up timepoint (6 weeks, 12 weeks, and 1 year), the interaction between group (TTI) and time, immobilization, open fracture, and Schatzker VI fracture pattern. Negative flexion estimates indicate lower flexion in the TTI group than in the iTPF group

The overall cohort had an average follow-up time of 1.25 years, and follow-up time was not significantly different between the TTI and iTPF groups (Table 1). Postoperative complications were compared among patients with at least 3 months of follow-up (*n* = 113). In this cohort, any reoperation occurred in 22 patients (19.5%) and was significantly more common in the TTI group than in the iTPF group (39.4% vs. 11.2%, *p* = 0.001) (Table 5). Nonunion was also significantly more common in the TTI cohort (15.2% vs. 2.5%, *p* = 0.022), as was culture-positive infection requiring reoperation (30.3% vs. 10.0%, *p* = 0.011) (Table 5). Symptomatic removal of hardware did not significantly differ between groups (6.1% vs. 3.8%, *p* = 0.628) (Table 5).

**Fig. 2:**
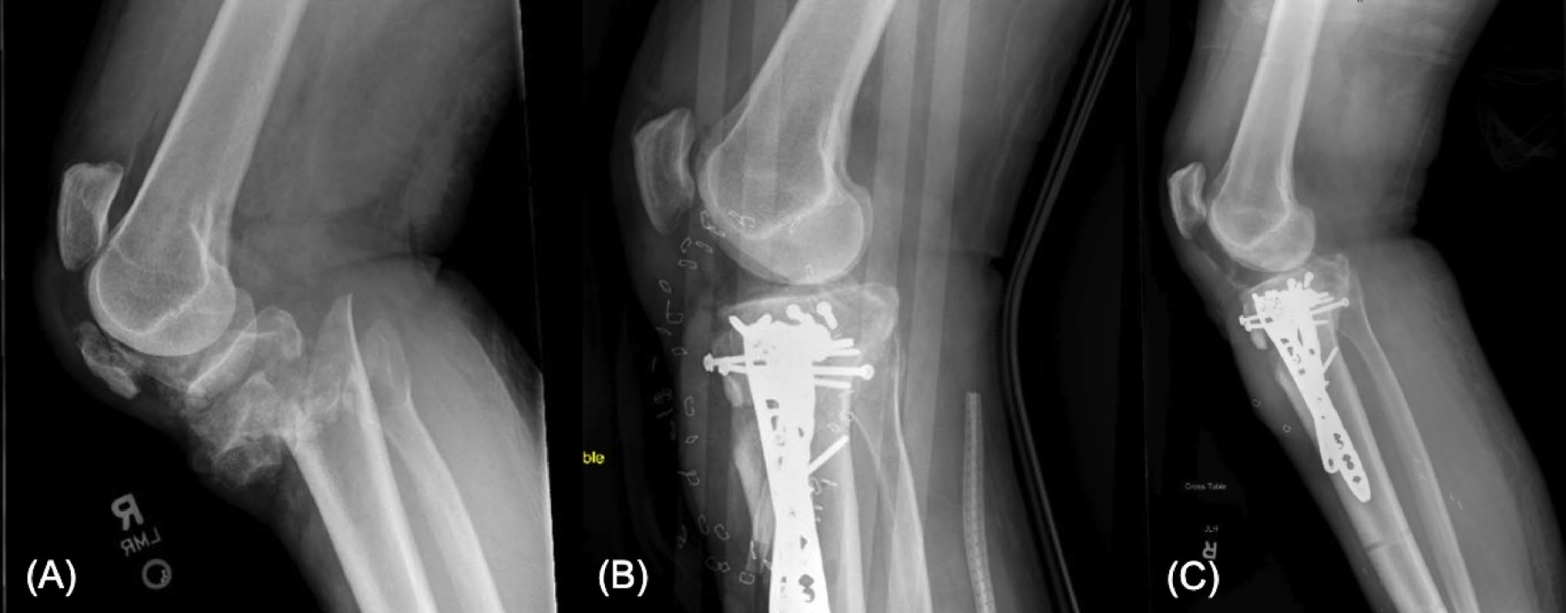
Representative case of tibial tubercle–involving tibial plateau fracture progressing to nonunion. **A** Preoperative lateral radiograph demonstrating displaced tubercle involvement with proximal tibial metaphyseal extension. **B** Immediate postoperative lateral radiograph after fixation. **C** Follow-up lateral radiograph showing fixation failure with persistent fragment displacement and progression to nonunion of the tubercle/metaphyseal segment. This case was later complicated by septic arthritis and hardware infection requiring irrigation and debridement with hardware removal

**Fig. 3:**
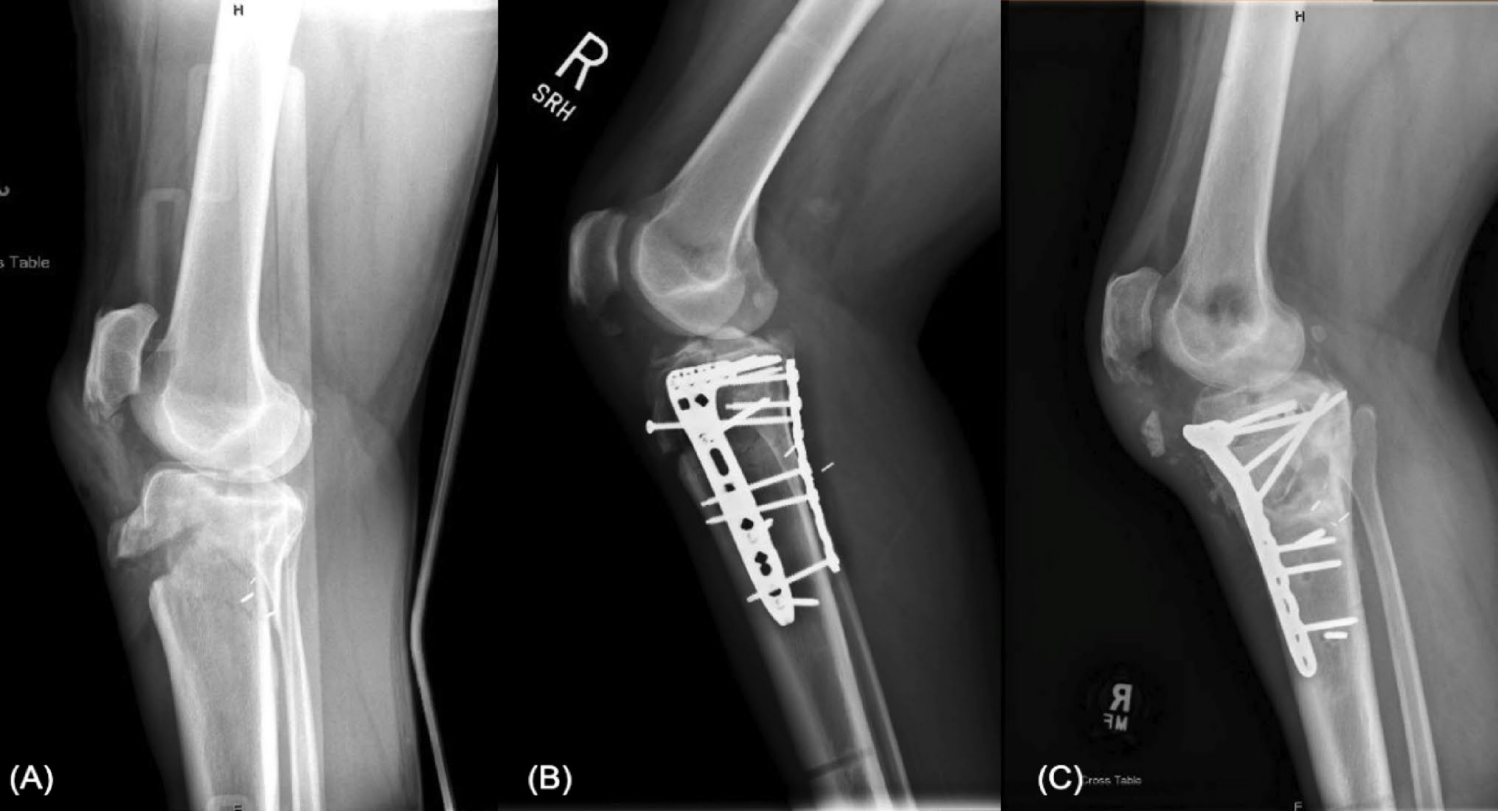
Representative case of fixation failure after operative treatment of a tibial tubercle–involving tibial plateau fracture. **A** Preoperative lateral radiograph. **B** Immediate postoperative lateral radiograph demonstrating the index fixation construct. **C** Follow-up lateral radiograph demonstrating construct failure and nonunion with the original hardware in situ, highlighting the significant mechanical strain on the extensor mechanism repair

In adjusted logistic regression limited to tibial tubercle involvement, open fracture, and Schatzker VI fracture pattern, TTI remained associated with higher odds of any reoperation (OR 3.95, 95% CI [1.36, 11.48]; *p* = 0.012) (Table 4). Open fracture (OR 3.33, 95% CI [0.57, 19.27]; *p* = 0.18) and Schatzker VI fracture pattern (OR 1.39, 95% CI [0.46, 4.26]; *p* = 0.561) were not statistically significant in the adjusted model Table 4). The nonunions included 1 patient that was indicated for surgery and preferred nonoperative management. Morphological analysis of the five nonunion cases in the TTI cohort using CT and plain film radiography cases revealed distinct anatomical patterns (Figs. 2 and 3). The majority of cases (*n* = 4) demonstrated tubercle and metaphyseal involvement characterized by a persistent fracture gap and lack of cortical bridging despite internal fixation. One case demonstrated a more complex failure pattern involving intra-articular extension into the proximal tibial metaphysis and nonunion of the lateral tibial plateau (Fig. 3).

**Table 4:** Adjusted logistic regression analysis of the predictors of any reoperation

Predictor	Adjusted OR	95% CI	*p* value
Tibial tubercle involvement	3.95	[1.36, 11.48]	0.012*
Open fracture	3.33	[0.57, 19.27]	0.18
Schatzker VI fracture	1.39	[0.46, 4.26]	0.561

Adjusted logistic regression for any reoperation was performed in the complication cohort and restricted to patients with ≥ 3-month follow-up. To reduce overfitting given the limited number of reoperation events (*n* = 22), the model was limited to tibial tubercle involvement, open fracture, and Schatzker VI fracture pattern

* indicates statistical significance at *p* ≤ 0.05

**Table 5:** Comparison of postoperative complications and reoperations between isolated TPF and TPF with tubercle involvement with ≥ 3 month follow-up

Outcome	Overall (*n* = 113)	iTPF (*n* = 80)	TTI (*n* = 33)	*p* value
Any reoperation	22 (19.5)	9 (11.2)	13 (39.4)	0.001*
Nonunion	7 (6.2)	2 (2.5)	5 (15.2)	0.022*
Culture-positive infection requiring reoperation	18 (15.9)	8 (10.0)	10 (30.3)	0.011*
Symptomatic removal of hardware	5 (4.4)	3 (3.8)	2 (6.1)	0.628

Surgical complications are reported for all patients with ≥ 3-month follow-up. Reoperation was defined as any unplanned return to the operating room related to the index fracture or its treatment

Culture-positive infection requiring reoperation refers to postoperative infection with a positive culture obtained at the time of reoperation

Symptomatic removal of hardware refers to reoperation for symptomatic implants. *P* values were calculated using Fisher exact tests

* indicates statistical significance at *p* ≤ 0.05. iTPF = isolated tibial plateau fracture, TTI = tibial tubercle involvement

## Discussion

In this retrospective study of tibial plateau fracture (TPF) patients, we found that the presence of a separate tibial tubercle fracture fragment was associated with higher rates of major postoperative complications, specifically nonunion and reoperation. In the reduced adjusted model, TTI remained a significant predictor of higher reoperation rates even when accounting for fracture severity and patient comorbidities. Additionally, our longitudinal mixed-effects analysis demonstrated that TTI was associated with worse flexion ROM, with significantly lower flexion at both 12 weeks and 1 year, whereas extension recovery did not differ between groups. These findings should be interpreted in the context of greater fracture severity and more frequent immobilization observed in the TTI cohort.

Our adjusted logistic regression analysis, which was limited to TTI, open fracture, and Schatzker VI fracture pattern to reduce overfitting, showed that TTI was associated with nearly a fourfold higher odd of reoperation (Table 4). Also, open fracture and Schatzker VI fracture patterns were not statistically significant in the reduced model, although this should not be interpreted as evidence that injury severity was unimportant. Rather, TTI may identify a subgroup with substantially higher surgical morbidity, while residual confounding from fracture complexity likely remains. The TTI group’s higher prevalence of Schatzker VI and open fracture patterns suggests that tubercle involvement may still function as a marker of higher-energy trauma [9, 10]. Additionally, the increased morbidity associated with TTI is likely multifactorial and related to both its underlying injury pathophysiology and the demands of surgical management. The unique complexity of TTI includes disruption of the knee extensor mechanism insertion and a challenging fracture morphology, which may require more invasive exposure and specialized fixation techniques to restore knee function. These factors likely contribute to higher rates of failure, nonunion, and reoperation.

The complication rates observed in the tibial tubercle cohort were consistent with the findings of the adjusted regression model (Tables 4 and 5). In the cohort with at least 3 months of follow-up, reoperation occurred in 39.4% of TTI fractures compared with 11.2% of iTPFs, and nonunion occurred in 15.2% versus 2.5%, respectively (Table 5). Culture-positive infection requiring reoperation was also more common in TTI (30.3% vs. 10.0%), whereas symptomatic removal of hardware did not significantly differ between groups (Table 5). Our radiographic review of these nonunion cases (Figs. 2 and 3) revealed that the majority involved the tubercle and metaphysis, though more complex patterns, including intra-articular extension into the proximal tibial metaphysis and nonunion of the lateral tibial plateau, were also present. The higher rate of nonunion in the TTI cohort likely stems from these complex morphological fracture patterns, which present technical challenges in achieving stable, anatomic fixation while simultaneously managing a compromised soft-tissue envelope [11]. Furthermore, while not reaching statistical significance for symptomatic removal of hardware, the TTI cohort experienced higher rates of several postoperative complications, including significantly higher rates of culture-positive infection requiring reoperation (Table 5). This trend toward higher morbidity likely reflects the more extensive surgical exposure and soft-tissue dissection that is required to achieve anatomic reduction of the tubercle fragment. These findings are consistent with Norris et al., where the proximal tibia’s vulnerable soft tissue envelope can only tolerate minimal surgical trauma; therefore, the extensive exposure required for anatomic reduction of tubercle fractures was associated with higher wound complications and deep infection rates approaching 6%, with septic arthritis occurring in 2.4% of cases [11]. Additionally, Papagelopoulos et al. and Berkson & Virkus highlight that these injuries are associated with more substantial soft tissue damage and that aggressive approaches to achieve definitive fixation methods can amplify postsurgical risks such as infection and nonunion [12, 13]. Furthermore, the complexity of TTI fixation frequently required additional implants, increasing the surgical burden [14]. These findings are reflected in the high complication rates (27.5%) reported by Lehane et al. for controlled tibial tubercle osteotomies, suggesting that traumatic TTI fractures faced even greater perioperative risks [15]. Ultimately, the pathophysiology of TTI integrated biomechanical instability, soft-tissue vulnerability, and surgical complexity, leads to significantly higher morbidity and major postsurgical complications even when controlling for open fracture status and Schatzker classification. Additionally, these outcomes were unlikely to be affected by monitoring bias, as no significant difference in follow-up duration existed between the two study cohorts.

Beyond complications, our mixed-effects analysis revealed that patients who sustained TPFs with tibial tubercle involvement had experienced significantly lower flexion ROM at both 12 weeks and 1 year after adjusting for immobilization, open fracture, and Schatzker VI fracture pattern (Table 3). This early difference should be interpreted cautiously because postoperative immobilization was substantially more common in the TTI cohort; the reduced 12-week and 1-year flexion likely reflects a combination of fixation strategy, greater injury severity, extensor mechanism involvement, and more restrictive postoperative management. Consequently, the present data cannot definitively determine whether the ROM deficit was primarily injury-related or protocol-driven. Nonetheless, these findings corroborate our initial hypothesis, suggesting that TTI does negatively influence postoperative ROM following TPF rehabilitation. The diminished flexion ROM observed at 12 weeks and 1-year postoperatively in the TTI group may stem from several factors. First, disruption of the knee extensor mechanism secondary to tubercle involvement can compromise its biomechanics, thereby increasing pain and interfering with early mobilization [6]. Second, our study showed a significant difference in immobilization status between groups. As we expected, patients with tibial tubercle involvement were significantly more likely to be placed in immobilization postoperatively compared to those without tubercle involvement in order to protect the extensor mechanism. Non-weight-bearing protocols were also more common with patients in the tubercle cohort compared to those without tibial tubercle involvement, though this difference did not reach statistical significance (Table 2). Although these described more restrictive postoperative management when the tibial tubercle is involved, the lack of significant differences in postoperative weight-bearing status may indicate variation in postoperative protocol preferences within our institution. Clinically, these results support closer surveillance of flexion recovery in patients with TTI and call for future studies examining whether fixation strategy or rehabilitation protocol can mitigate this persistent deficit.

Our results imply that these injury patterns may need closer postoperative monitoring and individualized rehabilitation that focuses on early protected weight-bearing, ROM exercises, and quadriceps strengthening to preserve flexion while simultaneously protecting the surgical fixation and integrity of the knee extensor mechanism [16]. Methods aimed at fixing tubercle anatomy, ensuring stable fixation, and restoring joint congruity are vital for improving knee function. These strategies help to decrease disruption of the knee extensor mechanism and reduce poorer outcomes in these patient populations [8, 17]. Ultimately, these findings highlight the need for individualized rehabilitation protocols and close clinical monitoring to mitigate the high-risk profile associated with these injuries, rather than predefined alternative surgical interventions.

Although both groups appeared to improve over time, our mixed-effects analysis demonstrated that patients with TTI continued to have significantly lower flexion at 12 weeks and 1 year compared to patients with iTPF. This suggests that while rehabilitation protocols emphasizing early, controlled ROM remain important for preventing stiffness and optimizing short-term recovery, later strengthening, gait training, and ROM work may still be insufficient to fully eliminate longer-term differences in flexion recovery between groups. For instance, longitudinal studies demonstrate that early deficits in knee ROM, gait, and function are common after tibial plateau fracture and that most patients experience meaningful improvements in joint kinematics, loading, and patient-reported outcomes over the first 6–12 months [18–21]. Bennett et al. showed that lower limb joint kinematics and activities of daily living scores improved up to six months, with only minor changes thereafter, indicating that intensive rehabilitation in the subacute phase may be important for maximizing long-term function [18]. Millar et al. found that joint loading and gait parameters plateau after six months, suggesting that the timing and intensity of rehabilitation during and after the initial bone-healing phase may influence final recovery [19]. Lastly, large cohort studies have likewise shown that pain, activities of daily living, sports participation, and quality of life continue to improve for years after injury [20, 21]. Taken together, these studies suggest that overall recovery after tibial plateau fracture can continue over time, but our findings indicate that patients with TTI may remain at risk for persistent flexion deficits despite that broader trajectory of improvement. Additionally, investigating which rehabilitation protocols are most optimal in the short-term, subacute, and long-term phases of postoperative TPF and TTI treatment through patient-recorded outcomes and knee function or activity level may be warranted.

Despite the sufficient body of research on the management of TPFs, few studies have investigated the management of TPFs with tubercle involvement [22]. Our findings highlight the significance and need for further research into treatment methods aimed at improving outcomes for these unique yet complex injuries. Moreover, biomechanical studies exploring the precise processes in which TTI affects knee biomechanics and postoperative ROM could help clarify these relationships [23]. For example, consider studies utilizing finite element models to assess knee joint loading, clinical data to address function of the knee extensor mechanism, and gait analysis to evaluate knee kinematics [6, 18, 23].

While studies exploring the tubercle’s role in knee biomechanics could help elucidate its specific mechanisms, careful assessment of the tibial tubercle, when involved in TPFs, is needed to reduce the risk of misinterpretation or misdiagnosis of this fracture type upon initial evaluation [22]. Tan et al. suggest that complex TPFs with tibial tubercle fractures can be overlooked due to fracture fragments obscuring the tubercle on imaging. They also mention that poor lateral knee radiographs can raise the risk of missing these fractures upon initial imaging studies. In light of these considerations, future research should focus on developing well-defined classification systems for TTI in TPFs. While this may not change the quality of the imaging itself, it can help in creating a more standardized framework for improving observer agreement between clinicians among clinical assessment of these fractures. Therefore, careful initial assessment and well-defined classification systems are both factors that can contribute to a lower risk of missed TTI on initial evaluation. Overall, having a more standard approach to evaluating these injury patterns can allow clinicians to make a more comprehensive treatment plan and prevent further complications that would reduce the chances of a successful recovery for patients with this injury type.

### Limitations

Certain limitations must be acknowledged when interpreting the results of this study. As a retrospective study, these findings may have introduced selection bias and limited the ability to obtain more comprehensive data regarding pain levels, functional outcomes, and long-term complications. Furthermore, while the overall cohort was 132 patients, the TTI subgroup (*n* = 38) and the total number of reoperation events in the adjusted complication cohort (*n* = 22) were relatively small for subgroup comparisons. The relatively small number of reoperation events limited model complexity and warrants cautious interpretation of the adjusted regression findings. Additionally, while significant between-group differences were observed for reoperation, nonunion, and culture-positive infection requiring reoperation, the event counts for individual complications remained moderate, which limits precision and may obscure additional clinically relevant differences in secondary outcomes.

Clinical interpretation is further limited by the lack of standardized postoperative protocols. Immobilization and rehabilitation were determined at the surgeon’s discretion, making it difficult to distinguish whether the observed 12-week and 1-year ROM deficits were primarily injury-related or protocol-driven. Although we addressed the effects of longitudinal postoperative knee ROM using mixed-effects models that accounted for repeated measurements and adjusted for immobilization, open fracture, and Schatzker fracture pattern, residual confounding from unmeasured factors remains possible. Specifically, variables such as fixation construct, soft-tissue injury severity, concomitant ligamentous or meniscal injury, and rehabilitation regimen. Additionally, ROM measurements were obtained retrospectively from routine clinical documentation and were incomplete across follow-up time points. Finally, because the study spanned 2014 to 2025, temporal changes in surgical techniques, implants, and perioperative care over the decade may have influenced outcomes.

In addition to these methodological constraints, a longer period of observation may be needed to obtain a more comprehensive spectrum of long-term consequences. Future research should evaluate whether the persistent flexion deficits observed in patients with TTI remain clinically meaningful beyond 1 year and whether they are associated with long-term complications or patient-reported functional limitations. This would provide greater insight into the lasting functional consequences of these injuries. Some of these outcomes may involve the development of post-traumatic osteoarthritis, persistent ROM limitations, or eventual need for total knee arthroplasty [2–4, 24, 25]. Additionally, studies should concentrate on implementing larger sample sizes, longer follow-up periods, and prospective designs to evaluate differences in outcomes based on surgical fixation methods as well as different postoperative rehabilitation strategies for TPFs with tibial tubercle involvement [25].

## Conclusion

In conclusion, our study demonstrates that tibial tubercle involvement in operatively treated tibial plateau fractures was associated with higher rates of major postoperative complications, specifically nonunion, reoperation and worse flexion recovery over time. In longitudinal mixed-effects analysis, patients with TTI demonstrated lower flexion at both 12 weeks and 1 year, whereas extension recovery did not differ between groups. These findings should be interpreted in the context of greater fracture severity and nonstandardized postoperative management in the TTI cohort. While these injuries are relatively uncommon, the presence of a tubercle fragment carries a high risk of adverse outcomes and increased overall morbidity, even when accounting for fracture severity. Ultimately, more research is essential to define surgical and rehabilitation plans that can improve functional outcomes in this high-risk patient population.

## Data Availability

The data used and generated in this study is not publicly available. However, the data may be available pending any required institutional agreements.
